# Treatment of Hepatocellular Carcinoma Using Endoscopic Ultrasound‐guided Radiofrequency Ablation: A Case Series

**DOI:** 10.1002/deo2.70171

**Published:** 2025-07-01

**Authors:** Yogesh Harwani, Shreya Butala, Varun Shukla, Anand Patel, Surbhi Dogra Jani

**Affiliations:** ^1^ Department of Gastroenterology Noble Gastro Hospital Ahmedabad India

**Keywords:** case series, endoscopic ultrasound, hepatocellular carcinomas, liver cirrhosis, radiofrequency ablation

## Abstract

Endoscopic ultrasound‐guided radiofrequency ablation (EUS‐RFA) is an emerging technique for treating pancreatic and neuroendocrine tumors in patients who are not candidates for surgery. However, there is limited evidence of EUS‐RFA in hepatic cancers. The present case series describes five elderly patients with compensated cirrhotic hepatocellular carcinomas (HCCs) treated locally using EUS‐RFA. Alpha‐fetoprotein levels were reduced in patients after 1 month of the procedure. Computed tomography analysis also reported a reduction in HCCs in patients postoperatively. Repeat computed tomography triple‐phase abdomen also showed complete radiological response to treatment in lesions <3 cm. None of the patients reported any procedural adverse event. EUS‐RFA offers safe ablation of <3 cm HCC lesions.

## 1 Introduction

Hepatocellular carcinoma (HCC), a significant contributor to the growing number of cancer patients, is among the leading causes of cancer‐related fatalities. Its primary risk factors include alcohol consumption, metabolic disorders, non‐alcoholic steatohepatitis (NASH), and hepatitis B/hepatitis C virus (HBV/HCV) infections [1]. For early‐stage HCC, radiofrequency ablation (RFA) is recommended as a curative treatment option [2]. While percutaneous RFA is effective for tumors ≤3.0 cm, its application is often limited by anatomical challenges, such as large interposing arteries, obesity, or unfavourable tumor locations (e.g., subcapsular, left, or caudate lobe) [2].

**FIGURE 1:**
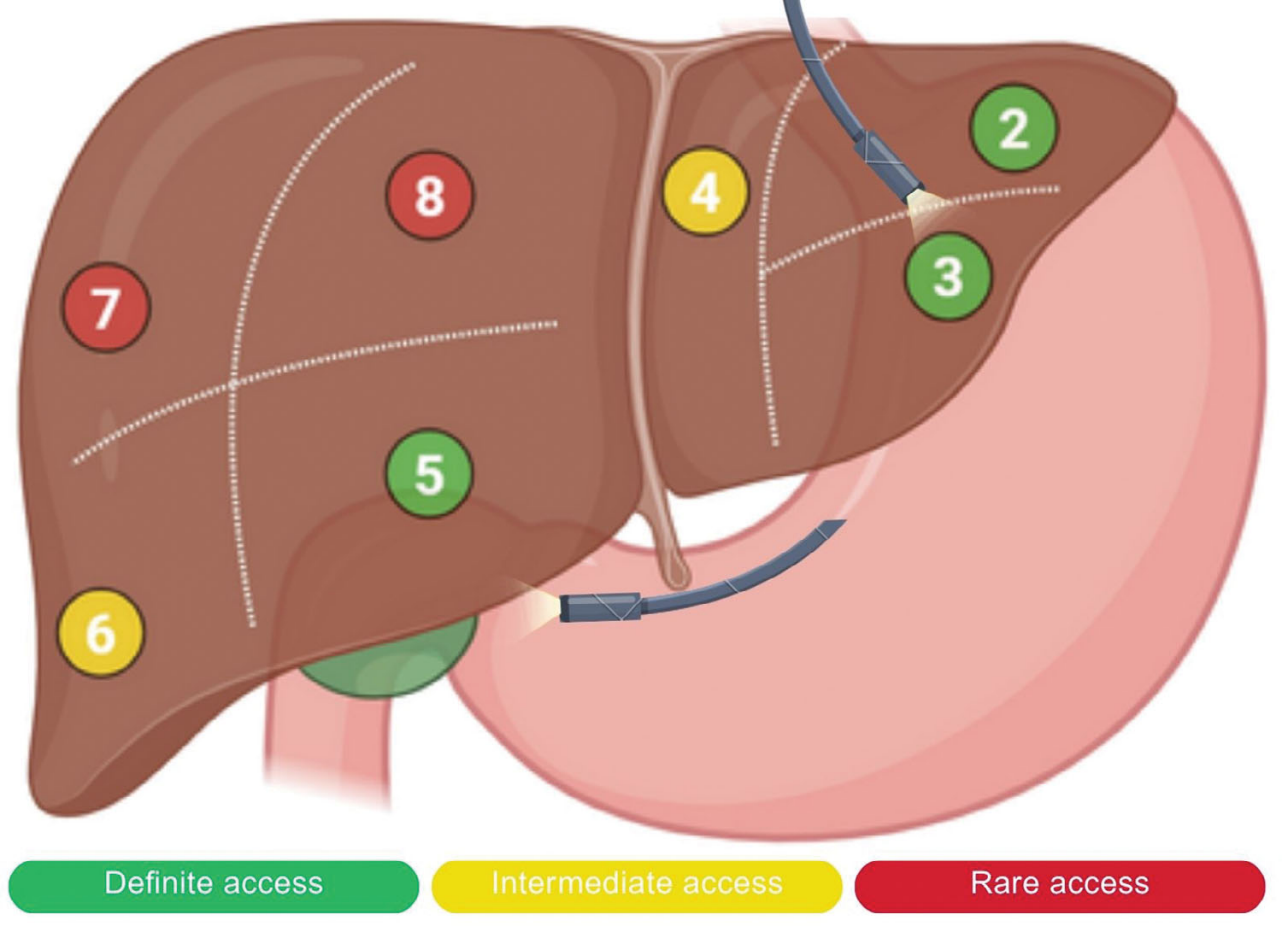
Diagrammatic representation of the feasibility of liver segments for endoscopic ultrasound‐guided radiofrequency ablation (EUS‐RFA) based on needle positioning.

To address these limitations, endoscopic ultrasound (EUS)‐RFA has emerged as a promising alternative (Figure 1) [3]. This technique has demonstrated success in treating pancreatic neuroendocrine tumors [4, 5], but its use for HCC remains limited. The present case series reports on five cirrhotic patients who underwent EUS‐RFA for HCC, along with their 1‐month follow‐up outcomes.

### 1.1 Case Report

Five HCC patients visited the Noble Gastro Hospital, India. EUS‐RFA was performed on the lesions as they were inaccessible via the percutaneous approach. The unconventional approach was considered as patients either presented with lesions in the left segments of the liver, or the gallbladder posed a potential obstruction to the RFA needle trajectory.

### 1.2 EUS‐RFA Procedure and Outcome Assessment

EUS‐RFA was carried out using a linear EUS endoscope under propofol anaesthesia. A 19G 10 mm monopolar needle (EUSRA RF electrode, Tae Woong Medical) (Figure S1) with a 140 cm long flexible electrode featuring an adjustable deployment length of up to 8 cm was used. The RFA needle was inserted into the lesion and ablated at 30 W for 15–20 s through a VIVA COMBO RF generator (Tae Woong/STARMED, Korea) (Figures S2 and S3). VIVA pump is attached to the RFA needle, which circulates saline through the needle for internal cooling, eliminating tissue charring by lowering the temperature at the needle tip. For larger lesions, the needle was inserted at multiple sites within the lesion. The needle tip was placed at the distal edge of the lesions and pulled toward the proximal end until the entire lesion was ablated. An abrupt increase in the real‐time tissue impedance acted as a signal for the termination of the ablation. The emergence of echogenic bubbles near the needle tip verified tissue necrosis.

Radiological response to EUS‐RFA was evaluated 1‐month post‐procedure using computed tomography (CT) triple‐phase abdomen scans. Pre‐ablation HCC was defined radiologically as non‐rim arterial phase hyperenhancement and non‐peripheral contrast washout on the portal venous and delayed phases. Complete radiological response was defined absence of arterial enhancement. Alpha‐fetoprotein (AFP) levels were also measured at the 1‐month follow‐up. Patients demonstrating a partial response after the 1‐month follow‐up were scheduled for repeat EUS‐RFA.

### 1.3 Investigations and Diagnosis

The clinical characteristics of the patients prior to treatment are summarized in Table S1. Case 1 was a 55‐year‐old male with compensated Hepatitis B‐related liver cirrhosis and HBV infection, presenting with a Child‐Turcotte‐Pugh (CTP) score of B. Imaging revealed a 1.2 × 1.2 cm lesion located in segment I. Case 2 was a 45‐year‐old male having compensated NASH‐related cirrhosis, with a single lesion sized 2.5 × 2.3 cm in segment VI. His CTP score was A, indicating preserved liver function. Case 3 was a 71‐year‐old male with decompensated NASH‐related liver cirrhosis and a CTP score of B. He had a history of ascites and presented with lesions in segment II (1.0 × 1.1 cm) and segment III (2.2 × 1.9 cm). Case 4 was a male aged 65 years, with compensated HBV‐related liver cirrhosis, and a CTP score of B. He exhibited multiple lesions in segments II (1.1 × 1.0 cm), III (1.0 × 0.9 cm), VIII (1.1 × 0.7 cm), and the junction of VII‐VIII (4 × 3.4 × 3.8 cm). Case 5 is a 67‐year‐old male with a CTP score of A, compensated liver cirrhosis with NASH, and multiple lesions, at segments II, VI, and III, measuring 1.3 cm, 1.4 cm, and 0.6 cm, respectively.

## 2 Treatment

**FIGURE 2:**
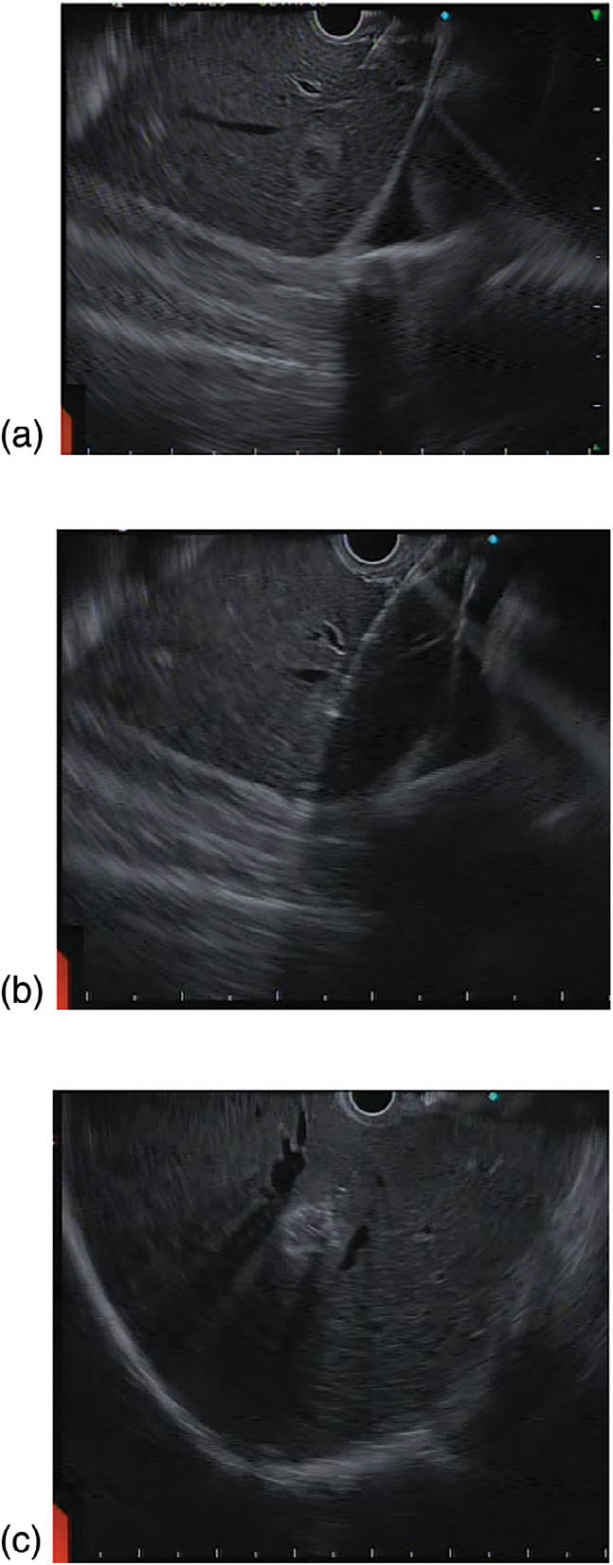
Intraoperative linear endoscopic ultrasound (EUS) taken during the radiofrequency ablation (RFA) procedure. (a) Hepatocellular carcinoma (HCC) in segment III of the liver; (b) EUSRA RFA probe within lesion; (c) Echogenic bubbles observed post‐RFA.

All patients successfully underwent the EUS‐RFA procedure as described above (Figure 2). Case 1 had three passes of the needle for ablation sessions at 30 W. Case 2 required six passes of the needle for ablation. Case 3 required four and six passes for ablation at lesions in segments II and III, respectively. Case 4 had two ablations for all the lesions. Likewise, case 5 also had multiple passes for ablation at 30 W.

### 2.1 Follow‐up and Outcomes

**FIGURE 3:**
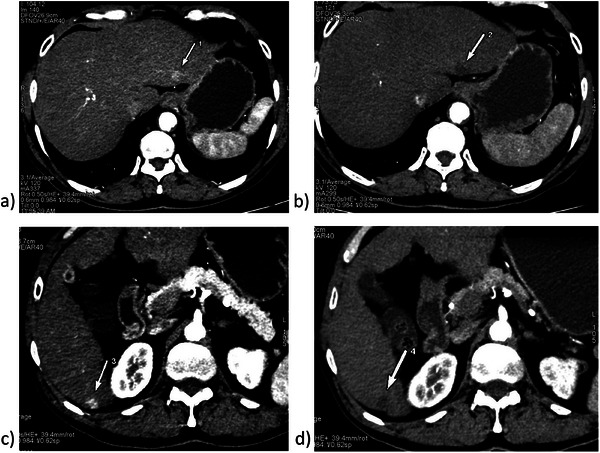
(a) Pre‐ablation hepatocellular carcinoma (HCC) lesion at segment II in case 5; (b) 1‐month post‐ablation HCC lesion at segment II in case 5; (c) Pre‐ablation HCC lesion at segment VI in case 5; (d) 1‐month post‐ablation HCC lesion at segment VI in case 5.

At the 1‐month follow‐up (Table 1 and Figure 3), a complete radiological response (absence of enhancement) was observed in four of the five patients (Cases 1, 2, 3, and 5). Case 4 demonstrated a partial response, given the difficulty in accessing segments VII and VIII. Nonetheless, Case 4 underwent a second round of EUS‐RFA, after which a complete radiological response was achieved.

**TABLE 1:** One‐month post‐procedural outcome of hepatocellular carcinoma (HCC) patients treated with endoscopic ultrasound‐guided radiofrequency ablation (EUS‐RFA).

	Pre‐procedure		1 Month Post‐procedure	
Case No.	Lesion size	AFP levels	Response	AFP levels
1	1.2 × 1.2 cm	31	Complete response	6.76
2	2.5 × 2.3 cm	24	Complete response	5.09
3	Segments II (L1: 1.0 × 1.1 cm) and III (L2: 2.2 × 1.9 cm)	42	Complete response in both lesions	32
4	Multiple lesions with sizes (L1 VII–VIII: 4 × 3.4 × 3.8 cm, L2 VIII: 1.1 × 0.7 cm, L3 II: 1.1 × 1.0 cm, L4 III: 1.0 × 0.9 cm)	20	L1 and L2: partial response. L3 and L4: complete response Repeat RFA performed at 1‐month follow‐up: CT showed a complete response	6.25
5	Segment II 1.3 cm, VI – 1.4 cm, III –0.6 cm	23.6	Complete response in all three lesions in segments II, III, and VI.	13

* RFA: Radiofrequency ablation; CT: computed tomography; AFP: Alpha‐fetoprotein.

No immediate complications or adverse events were noted post‐EUS‐RFA. The post‐procedural hospital course was uneventful, and all patients were discharged.

## 3 Discussion

Based on the complete response observed in all patients in our study, our findings suggest that EUS‐RFA is a safe and effective approach for the treatment of HCC patients, specifically in those who are not suitable for surgical resection. This is a notable finding, as less than 20% of HCC patients qualify for surgery due to frequent presentation with cirrhosis or chronic liver disease [2]. For patients at high surgical risk, EUS‐RFA offers a minimally invasive and potentially effective alternative for treating HCC lesions and associated symptoms [5].

EUS‐RFA provides greater precision in targeting HCC nodules by allowing real‐time visualization of vascular structures, which can help minimize the risk of thermal injury [6]. This technique is particularly beneficial for tumors in anatomically difficult locations, which are challenging to access via percutaneous methods [7]. However, this approach is not without risks. Patients with cirrhosis and prior abdominal surgery may be at an increased risk of complications, and additional studies are needed to compare the safety profile of EUS‐RFA with other ablative techniques.

Despite its potential, EUS‐RFA remains understudied, with most available data limited to case reports [3, 8, 9, 10]. In 2018, Attili et al. described a patient with HCV‐related HCC in segment VIII successfully treated with EUS‐RFA [8]. Recently, a patient aged 64 years presenting with HCC within the caudate lobe and a 48‐year‐old patient with a 2 × 1.6 × 1.6 cm lesion were successfully treated with EUS‐RFA [9, 10]. These cases highlight the growing clinical adoption of EUS‐RFA for tumors that are difficult to reach percutaneously.

Our case series advances prior clinical reports by presenting novel evidence on the feasibility and effectiveness of EUS‐RFA in five patients with HCC and cirrhosis. The case series reveals that EUS‐RFA was effective in achieving complete ablation for tumors <3 cm in four out of five patients. In one case with a partial response, repeat ablation was performed successfully. However, given the limited follow‐up period and number of cases, further studies with larger cohorts should evaluate long‐term outcomes, recurrence rates, and comparative effectiveness between percutaneous ablation and surgical resection. Additionally, future studies should investigate the safety profile of EUS‐RFA in patients with advanced cirrhosis and portal hypertension, as these populations may have a higher risk of complications.

## Conflicts of Interest

The authors declare no conflicts of interest.

## Supporting information



Supporting File: deo270171‐sup‐0001‐figureS1.jpg

Supporting File: deo270171‐sup‐0002‐figureS2.jpg

Supporting File: deo270171‐sup‐0002‐figureS3.jpg

Supporting File: deo270171‐sup‐0004‐tableS1.docx

Supporting File: deo270171‐sup‐0005‐videoS1.mp4

## Data Availability

None
