# Supplementary figures and images for: Treatment of Hepatocellular Carcinoma Using Endoscopic Ultrasound‐guided Radiofrequency Ablation: A Case Series
Source: DEN Open. 2025 Jul 1;6(1):e70171. doi: 10.1002/deo2.70171 (PMC12210139; doi:10.1002/deo2.70171)

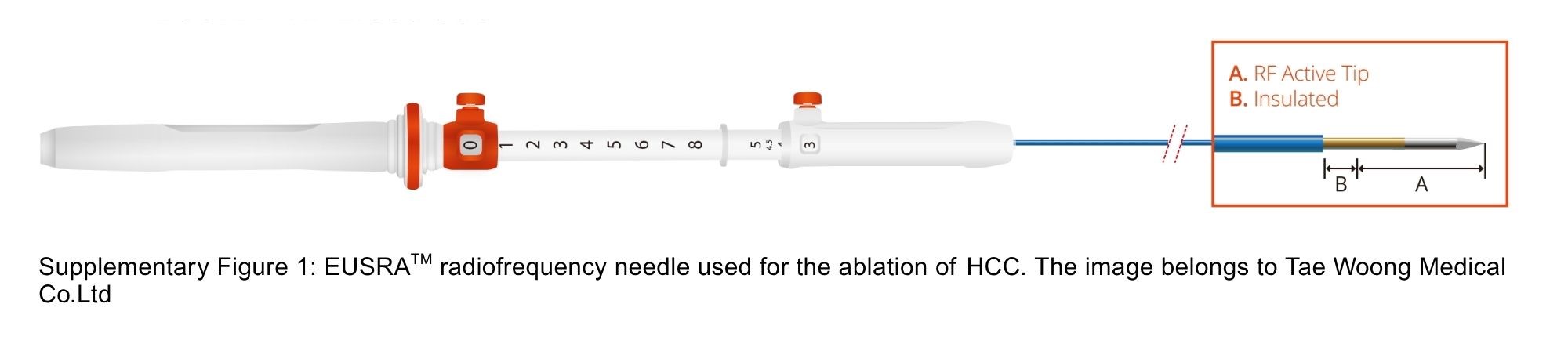

Supplement: Supplementary file 1 — Supporting File: deo270171‐sup‐0001‐figureS1.jpg [file DEO2-6-e70171-s003.jpg]

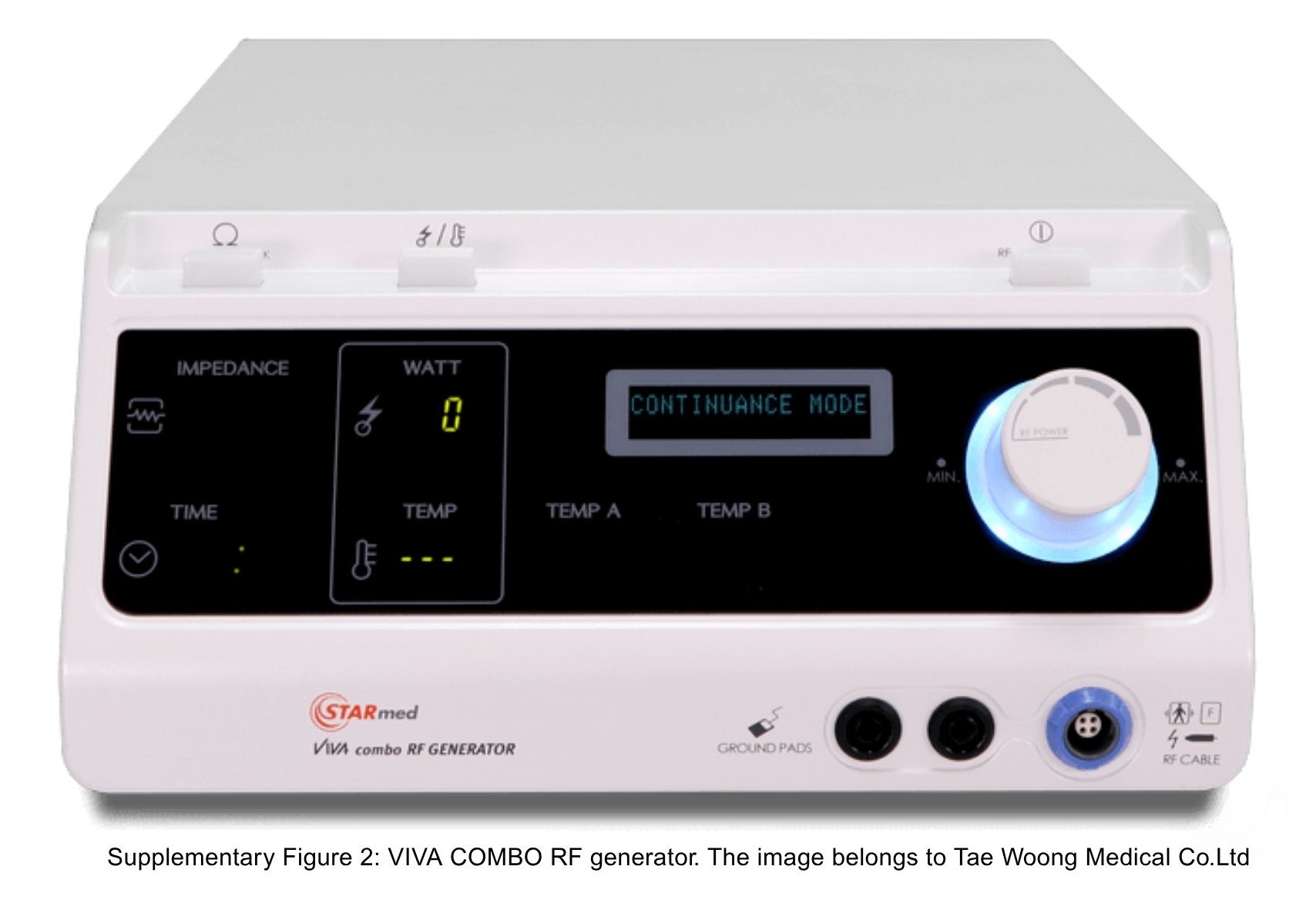

Supplement: Supplementary file 2 — Supporting File: deo270171‐sup‐0002‐figureS2.jpg [file DEO2-6-e70171-s005.jpg]

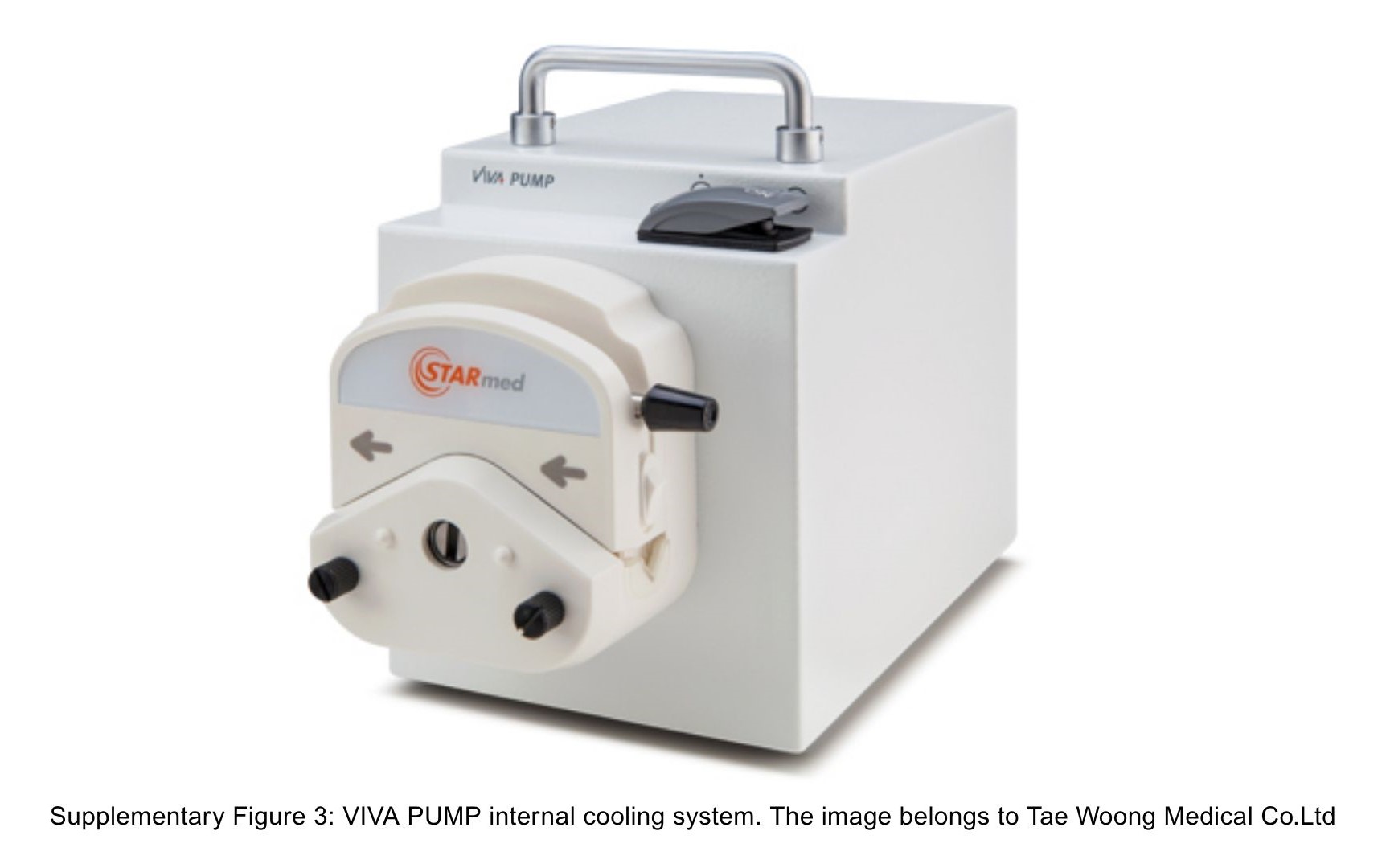

Supplement: Supplementary file 3 — Supporting File: deo270171‐sup‐0002‐figureS3.jpg [file DEO2-6-e70171-s002.jpg]
