# Supplementary material for: Treatment of Hepatocellular Carcinoma Using Endoscopic Ultrasound‐guided Radiofrequency Ablation: A Case Series
Source: DEN Open. 2025 Jul 1;6(1):e70171. doi: 10.1002/deo2.70171 (PMC12210139; doi:10.1002/deo2.70171)
Supplement: Supplementary file 4 — Supporting File: deo270171‐sup‐0004‐tableS1.docx [file DEO2-6-e70171-s001.docx]

Supplementary Table 1: Summary of case presentation of all 5 patients

| **Cases** | **Age** | **Gender** | **Etiology** | **Cirrhosis** | **CTP score** | **Segment of lesion location** |
| --- | --- | --- | --- | --- | --- | --- |
| 1 | 55 | M | HBV | Decompensated | B | I |
| 2 | 51 | M | NASH | Compensated | A | III |
| 3 | 71 | M | NASH | Decompensated | B | II and III |
| 4 | 65 | M | HBV | Compensated | B | Junction of VII-VIII, VIII,II,III |
| 5 | 67 | M | NASH | Compensated | A | II, III, VI |
